# Endovascular Thrombectomy Versus Best Medical Management Beyond 24 Hours From Last Known Well in Acute Ischemic Stroke Due to Large Vessel Occlusion

**DOI:** 10.1161/SVIN.122.000790

**Published:** 2023-04-28

**Authors:** Permesh Singh Dhillon, Waleed Butt, Tudor G. Jovin, Anna Podlasek, Norman McConachie, Robert Lenthall, Sujit Nair, Luqman Malik, Kailash Krishnan, Robert A. Dineen, Timothy J. England

**Affiliations:** ^1^ Interventional Neuroradiology Queens Medical Centre, Nottingham University Hospitals NHS Trust Nottingham UK; ^2^ Radiological Sciences, Mental Health & Clinical Neuroscience University of Nottingham Nottingham UK; ^3^ Interventional Neuroradiology Queen Elizabeth Hospital, University Hospitals Birmingham NHS Trust Birmingham UK; ^4^ Neurology Cooper University Hospital Camden NJ; ^5^ Tayside Innovation Medtech Ecosystem (TIME) University of Dundee UK; ^6^ Stroke Medicine Queens Medical Centre, Nottingham University Hospitals NHS Trust Nottingham UK; ^7^ NIHR Nottingham Biomedical Research Centre University of Nottingham Nottingham UK; ^8^ Stroke, Mental Health and Clinical Neuroscience, School of Medicine University of Nottingham Derby UK; ^9^ Stroke University Hospitals of Derby and Burton NHS Foundation Trust Derby UK

**Keywords:** ischemia, occlusion, stroke, thrombectomy

## Abstract

**Background:**

The safety and efficacy of endovascular thrombectomy (EVT) in patients with acute ischemic stroke due to large vessel occlusion presenting beyond 24 hours from last known well (LKW) remains undetermined.

**Methods:**

In this single center study, we identified patients with large vessel occlusion who were eligible for EVT based on noncontrast computed tomography (CT)/CT angiography (without CT perfusion or magnetic resonance imaging) using an Alberta Stroke Program Early CT Score of ≥5, National Institutes of Health Stroke Scale of ≥6, and presenting beyond 24 hours from LKW, between January 2018 and March 2022. During the study period, EVT service limitations meant patients eligible for EVT presenting outside service hours, routinely received best medical management (BMM). Functional and safety outcomes were compared between patients receiving EVT or BMM following multivariable adjustment for age, baseline stroke severity, Alberta Stroke Program Early CT Score, time from LKW, IV thrombolysis, and clot location.

**Results:**

Among 35 patients presenting beyond 24 hours from LKW and eligible for EVT, 19 (54%) were treated with EVT and 16 (46%) with BMM. Alberta Stroke Program Early CT Score were similar across both groups (EVT: 7 [6.75–8] versus BMM: 7 [6–8]), but not the baseline National Institutes of Health Stroke Scale (EVT: 17 [11–19.5] versus BMM: 20 [9.75–26]). No significant difference was observed between the EVT and BMM groups in the symptomatic intracranial hemorrhage (5.3% versus 0%; *P*=0.28) or mortality (26.3% versus 37.5%; *P*=0.42) rates, respectively. The modified Rankin scale at 90 days (adjusted common odds ratio [OR], 1.94; [95% CI 0.42–8.87]; *P*=0.39) and functional independence rate, although numerically higher in the EVT group compared with the BMM group (modified Rankin scale≤2; 36.9% versus 18.8%; adjusted OR, 4.34; [95% CI 0.34–54.83]; *P*=0.25), were not significantly different. 94.7% of patients treated with EVT achieved successful reperfusion (modified thrombolysis in cerebral infarction 2b–3).

**Conclusion:**

In routine clinical practice, EVT beyond 24 hours from LKW appears safe and feasible, when performed in patients with acute ischemic stroke who were deemed eligible for EVT by noncontrast CT /CT angiography alone. A large collaborative randomized trial assessing the efficacy of EVT beyond 24 hours is warranted. Our findings provide a basis for the sample size estimate for an adequately powered trial.

**Nonstandard Abbreviations and Acronyms**

ASPECTS: Alberta Stroke Program Early CT Score
BMM: best medical management
CTA: computed tomography angiography
EVT: endovascular thrombectomy
mRS: modified Rankin scale
NCCT: noncontrast computed tomography

**Clinical Perspective**

- We evaluated the safety and functional outcomes of patients with acute ischemic stroke due to proximal large vessel occlusion, presenting beyond 24 hours from last known well who were eligible for endovascular thrombectomy (EVT) selected with noncontrast computed tomography (CT)/CT angiography alone (without CT perfusion or magnetic resonance imaging), by comparing patients eligible for EVT treated with EVT (presenting during regular working hours) and a comparable group treated with best medical management only (presenting outside EVT service hours).
- Patients treated with EVT had nonsignificantly higher rates of functional independence and symptomatic intracranial hemorrhage, but with lower rates of mortality, compared with patients treated with best medical management only.
- Our findings suggest that EVT appears safe and feasible when performed in patients with acute ischemic stroke with proximal large vessel occlusion selected without CT perfusion or magnetic resonance imaging beyond 24 hours from last known well, and provide a basis for the sample size estimate for an adequately powered trial in this very late window.

The DAWN (DWI or CTP Assessment With Clinical Mismatch in the Triage of Wake‐Up and Late Presenting Strokes Undergoing Neurointervention With Trevo) and DEFUSE‐3 (Endovascular Therapy Following Imaging Evaluation for Ischemic Stroke) randomized controlled trials demonstrated treatment benefit of endovascular thrombectomy (EVT) for large vessel occlusion in acute ischemic stroke for patients with a suitable mismatch identified on computed tomography perfusion (CTP) or magnetic resonance imaging (MRI), and presenting between 6 and 16 or 24 hours from the onset of stroke or last known well (LKW).^1^, ^2^ The findings indicate that salvageable tissue in patients with good collateral circulation can persist well beyond 6 hours.^3^, ^4^, ^5^

However, there are limited data on the clinical outcomes in patients treated with EVT beyond 24 hours from LKW. Only a few studies of modest sample sizes have reported outcomes in this time window, while assessment of the absolute treatment benefit has been limited due to the lack of comparison to a suitably matched control group of patients who did not undergo EVT.^6^, ^7^, ^8^, ^9^, ^10^

Hence, we sought to investigate the safety and functional outcomes of patients selected with noncontrast computed tomography/computed tomography angiography (NCCT/CTA) alone (without CTP or MRI) beyond 24 hours from LKW, by comparing patients eligible for EVT treated with EVT and a comparable group treated with best medical management (BMM) only.

## Methods

### Ethics

This study was registered with and approved by the local institutional board review (Ref ID: 22‐158C). Retrospective patient consent was not required for this study which was conducted in a deidentified manner. Data that support the findings of this study are available upon reasonable request.

### Data Source and Study Design

We performed a retrospective analysis of 2 prospectively defined cohorts according to the Strengthening the Reporting of Observational Studies in Epidemiology (STROBE) guidelines, on prospectively collected SSNAP (Sentinel Stroke National Audit Programme) registry^11^ and local data for all adult (≥18 years) admissions with an acute ischemic stroke who presented directly or were transferred to a single tertiary EVT capable neuroscience center in the United Kingdom, between January 1, 2018 and March 31, 2022. The selection of patients eligible for EVT in clinical practice was based on our institution's protocol of the initial imaging performed (NCCT and/or dual‐phase CTA) regardless of the time window from stroke onset. The inclusion criteria for this study included: (i) occlusion of the intracranial internal carotid artery, M1 or proximal M2 segment of the middle cerebral artery, (ii) premorbid disability using the modified Rankin scale (mRS) of 0 to 2, (iii) baseline stroke severity National Institutes of Health Stroke Scale of 6 and above, (iv) baseline Alberta Stroke Program Early CT Score (ASPECTS) of 5 and above, and (v) presentation from LKW of beyond 24 hours. Patients with a presentation from LKW of less than 24 hours, those with an ASPECTS score of 0–4, or posterior circulation stroke were excluded. The mRS data at discharge were extrapolated (assuming no further improvement or worsening) for those with missing 90‐day mRS scores (n=5).

Patients were divided into 2 groups according to the treatment received: (i) EVT and (ii) BMM. At our institution over the study period, EVT treatment was only offered to eligible patients between 8 am and 6 pm Monday to Friday due to limited capacity. Patients who presented out of hours or on weekends, including those eligible for EVT treatment, were treated with BMM only. This limited EVT service availability allows a comparison of EVT and BMM in patients who meet the same inclusion criteria, in which selection based on physician‐related bias is significantly reduced.

Due to the stroke pathway limitations during the study period, patients could not be transferred to another center offering EVT outside service hours. A small cohort of patients who presented out of hours, but remained eligible using the same clinical and imaging criteria the next day following repeat neuroimaging, were offered EVT treatment. A minority of patients may have also presented under 4.5 hours from stroke onset (out of hours) and were treated with intravenous thrombolysis treatment following late repeat neuroimaging. Patients who received BMM at our institution were admitted to a dedicated hyperacute stroke unit and were treated according the National Institute for Health and Care Excellence (NICE) guidelines,^12^ which included 300 mg aspirin on admission if they were ineligible for intravenous‐tissue plasminogen activator treatment, adequate blood pressure, and blood glucose control.

### Outcome Measures

The functional outcome (including functional independence (mRS≤2)) was assessed with the mRS score at 90 days. Safety outcomes were mortality at 90 days and symptomatic intracranial hemorrhage defined according to European Collaborative Acute Stroke Study (ECASS) II classification^13^ as any intracranial hemorrhage with an increase of the National Institutes of Health Stroke Scale score of ≥4 within 24 hours or death. Procedural outcome was successful reperfusion (modified thrombolysis in cerebral infarction score of 2b–3) at the end of EVT, as retrospectively assessed by an interventional neuroradiologist.^14^ Baseline clinical data were retrieved from the prospective stroke registry and functional outcome measure (mRS) was prospectively assessed by a member of the Stroke team/physician, or by a trained specialist nurse during a routine clinical follow‐up. ASPECTS, collateral circulation status were retrospectively assessed by 2 trained neuroradiologists, blinded to the treatment allocation. Intraclass correlation coefficient was calculated as a measure of interrater reliability between the ASPECTS obtained prospectively for treatment purposes and the ASPECTS following retrospective review.

### Statistical Analysis

Study characteristics were summarized using descriptive statistics for patient demographics, clinical characteristics, comorbidities, and time metrics. Comparisons of baseline variables were made using the Chi‐square, or Student's t‐test, wherever applicable.

Analyses of the outcome measures used ordinal logistic regression for the full‐scale mRS as primary outcome and binary regression analysis for the remaining dichotomized clinical outcomes. Multivariable regression analysis was conducted, adjusted for variables of clinical relevance: age, baseline stroke severity (National Institutes of Health Stroke Scale), ASPECTS, clot location, presentation time from LKW, and prior intravenous thrombolysis use. A sensitivity analysis of the functional outcome was also performed, excluding patients with a missing mRS at 90 days. Two‐tailed *P* value of <0.05 was considered statistically significant. Analyses were conducted using StataSE 17.1.

## Results

**Figure 1:**
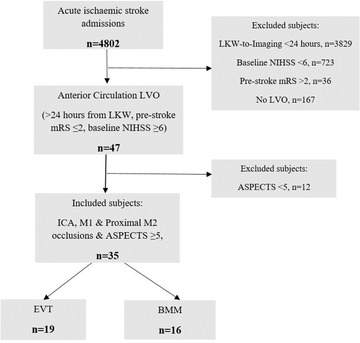
**Flow chart of the patient inclusion, exclusion and outcome data for patients identified as eligible for EVT with noncontrast CT/CT angiography only**, **and treated with EVT or BMM beyond 24 hours from last known well**. ASPECTS indicates Alberta Stroke Program Early CT Score; BMM, best medical management; EVT, endovascular thrombectomy; ICA, intracranial internal carotid artery; LVO, large vessel occlusion; MCA, middle cerebral artery; mRS, modified Rankin scale; n, number of events; and NIHSS, National Institutes of Health Stroke Scale.

During the study period, a total of 4802 patients with acute ischemic stroke presented directly to a single EVT‐capable neuroscience center. We included 35 patients presenting after 24 hours from LKW and assessed as being eligible for EVT, of whom 19 (54%) were treated with EVT, and 16 (46%) were treated with BMM only (Figure 1). Eleven patients (31%) had a witnessed stroke onset, the remainder were documented as LKW. Compared with the BMM cohort, patients treated with EVT were younger (69.3±14.9 versus 78.3±9.1 years) and had a marginally lower baseline stroke severity (National Institutes of Health Stroke Scale) (median 17 [11–20.5] versus 20 [9.75–26]) (Table 1). No significant differences were observed in the remaining baseline characteristics, including the baseline ASPECTS (median 7 [6.75–8] versus 7 [6–8]), between the 2 groups (Table 1), with 2 patients in each group scoring a baseline ASPECTS of 5. The mean LKW time‐to‐presentation in the EVT cohort was 34.6±11.5 hours compared with 37.5 ± 16.6 hours in the BMM group. No patients eligible for EVT were treated with EVT outside of the regular work hours during the study period. There was good interrater reliability (intraclass correlation coefficient, 0.69; [95% CI 0.58–0.82]) between the ASPECTS obtained for treatment purposes and following the retrospective review.

**Table 1:** Table of Characteristics Comparing Patients Identified as Eligible for EVT With Noncontrast CT/CT Angiography Only and Treated With EVT or BMM Beyond 24 Hours From Last Known Well

Feature	EVT n (%) Median (IQR) or mean±SD	BMM n (%) Median (IQR) or mean±SD	*P* value
Sociodemographics			
Sample size	19	16	–
Sex (male)	9 (47.3)	4 (25.0)	0.17
Age	69.3±14.9	78.3±9.1	0.044^†^
**Baseline characteristics**			
NIHSS on admission	17 (11–20.5)	20 (9.75–26)	0.30
Prestroke disability (mRS)	0 (0–0)	0 (0–0)	0.45
ASPECTS	7 (6–8)	7 (6.75–8)	0.77
ICA clot	6 (31.6)	2 (12.5)	0.31
M1 clot	10 (52.6)	9 (56.3)	0.92
Proximal M2 clot	3 (15.8)	5 (31.2)	0.35
Moderate‐good collaterals (Tan 2–3)^*^	18 (94.7)	9/10 (90.0)	0.62
IV thrombolysis	3 (15.7)	0 (0.0)	0.10
Witnessed stroke onset	5 (26.3)	6 (37.5)	0.59
Time delay from LKW (h)	34.6±11.5	37.5±16.6	0.54
**Comorbidities**			
Hypertension	10 (52.6)	11 (68.7)	0.39
Diabetes mellitus	2 (10.5)	1 (6.3)	0.52
Atrial fibrillation	4 (21.0)	7 (43.7)	0.17
Prior stroke/TIA	2 (10.5)	3 (18.7)	0.79

ASPECTS indicates Alberta Stroke Program Early CT Score; BMM, best medical management; EVT, endovascular thrombectomy; ICA, internal carotid artery; IQR, interquartile range; LKW, last known well; M1, first segment of the middle cerebral artery; M2, second segment of the middle cerebral artery; mRS, modified Rankin scale; n, number of events; NIHSS, National Institutes of Health Stroke Scale; TIA, transient ischemic attack.

^*^ Available data: N=10 BMM group.

^†^ Indicates statistical significance.

### Outcomes

**Figure 2:**
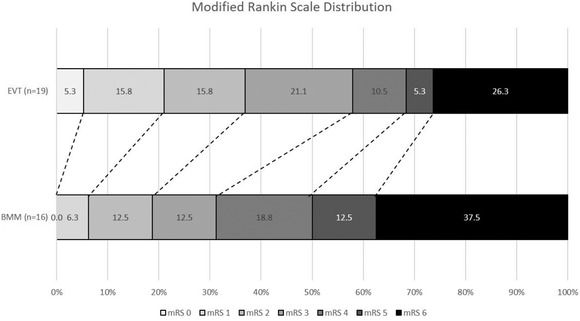
**Distribution of the modified Rankin scale (0 – no disability to 5 – severe disability and 6 – death) at 90 days comparing patients with large vessel occlusion selected** **with noncontrast CT/CT angiography only and treated with endovascular thrombectomy (EVT) or best medical management (BMM) beyond 24 hours from last known well**.

There was no significant difference in the functional outcomes at 90 days (adjusted common odds ratio [OR], 1.94; [95%CI 0.42–8.87]; *P*=0.39) among patients selected with NCCT/CTA alone and treated with EVT beyond 24 hours from LKW, compared with the BMM group (adjusted common OR, 1.94; [95%CI 0.42–8.87]; *P*=0.39). Patients in the EVT group achieved numerically higher rates of functional independence, without reaching statistical significance (mRS≤2; 36.9% versus 18.8%; adjusted OR, 4.34; [95%CI 0.34–54.83]; *P*=0.25) (Table 2, Figure 2). No significant difference was observed between the EVT and BMM groups in the remaining outcomes measures of symptomatic intracranial hemorrhage (5.3% versus 0%; *P*=0.28) or mortality at 90 days (26.3% versus 37.5%; *P*=0.42), respectively (Table 2). 94.7% of patients achieved successful reperfusion following EVT. No significant difference in functional outcome was demonstrated between the groups following sensitivity analysis of patients with a missing 90‐day mRS excluded (adjusted common OR,2.81; [95%CI 0.77–10.18]; *P*=0.14) and (mRS≤2; 38.8% versus 14.3%; adjusted OR, 3.81; [95%CI 0.65–22.45]; *P*=0.13).

**Table 2:** Table of Outcomes Comparing Patients Identified as Eligible for EVT With Noncontrast CT/CT Angiography Only and Treated With EVT or BMM Beyond 24 Hours From Last Known Well

Outcome measures	EVT (n=19) N (%)	BMM (n=16) N (%)	EVT vs BMM			
			Unadjusted OR (95% CI)	*P* value	Adjusted aOR (95% CI)^*^	*P* value
mRS at 90 d (ordinal)	3 (2–5.5)	4.5 (3–6)	2.39 (0.71–8.00)	0.15	1.94 (0.42–8.87)	0.39
mRS ≤2 at 90 d	7 (36.9)	3 (18.8)	2.52 (0.53–12.07)	0.24	4.34 (0.34–54.83)	0.25
mTICI 2b–3	18 (94.7)	–	–	–	–	–
sICH	1 (5.3)	0 (0)	1.76 (0.14–21.47)	0.65	6.08 (0.23–160.53)	0.28
Mortality (90 d)	5 (26.3)	6 (37.5)	0.59 (0.14–2.50)	0.47	0.20 (0.00–10.13)	0.42

aOR indicates adjusted odds ratio; ASPECTS, Alberta Stroke Program Early CT Score; BMM, best medical management; EVT, endovascular thrombectomy; mRS, modified Rankin scale; mTICI, modified thrombolysis in cerebral infarction; N, number of patients; NIHSS, National Institutes of Health Stroke Scale; and sICH, symptomatic intracranial hemorrhage.

^*^Adjusted multivariate analysis for age, baseline NIHSS, ASPECTS, onset‐to‐imaging time, use of intravenous thrombolysis, and clot location.

## Discussion

This real‐world study provides novel data on the clinical outcomes, among those who were eligible for EVT and treated with EVT compared to BMM alone, following imaging selection with NCCT/CTA only (without CTP or MRI) beyond 24 hours from LKW. Compared with patients treated with BMM only, a nonsignificantly higher proportion in functional independence rates (mRS≤2) at 90 days (36.9% versus 18.8%) was observed in patients treated with EVT. Although the incidence of symptomatic intracranial hemorrhage was numerically higher in the EVT group, the mortality rate at 90 days in the EVT group was nonsignificantly lower than the BMM group. Overall, compared with those treated with BMM only, this suggests that performing EVT beyond 24 hours from LKW appears safe and feasible.

It has been proposed that salvageable penumbra may persist beyond 24 hours, particularly in “slow progressors” with a small infarct core and tenacious collateral supply.^3^, ^4^ This cohort of patients with a persistent penumbral profile are more likely to have a poor functional outcome without reperfusion therapy.^5^ However, the safety and efficacy of EVT beyond 24 hours remains undetermined, as current guidelines recommend EVT treatment for eligible patients only up to 24 hours from LKW.^15^, ^16^ Previous case series or retrospective studies of modest sample sizes have attempted to assess the effectiveness of EVT beyond 24 hours from LKW in patients selected with advanced neuroimaging (CTP or MRI), some mirroring the inclusion criteria of the late window trials.^6^, ^7^, ^8^, ^9^ A previous single center study reported significantly improved functional outcome at 90 days (but not functional independence) in this very late window among patients treated with EVT compared with those treated with BMM alone.^8^ Although the rates of functional independence have been reported in the range of 41% to80%, the paucity of a control group of BMM patients in other prior studies has restricted the assessment of the absolute treatment efficacy.^6^, ^7^, ^9^ Furthermore, no prior study has evaluated the treatment efficacy and safety among patients selected for EVT eligibility based on NCCT/CTA imaging alone beyond 24 hours from LKW. While the analyses in our study were likely underpowered, our findings suggest that the use of NCCT/CTA alone might be a feasible option to select patients for EVT beyond 24 hours from LKW, and that EVT remains a safe and viable option for patients with a large vessel occlusion in this EVT window, as evidenced by the 18% point difference in the functional independence rate with comparable safety outcomes between the EVT and BMM groups. The proportion of patients treated with EVT who achieved functional independence (37%) is similar to that reported in the MR CLEAN LATE trial (39%) that assessed eligibility for EVT without CTP/MRI in patients presenting between 6 and 24 hours from LKW (results presented at the World Stroke Congress 2022).^17^ Furthermore, the findings in this study provide a basis for the sample size estimate in planning an adequately powered trial, with an estimated 95 patients per treatment group would have 80% power to detect an 18% proportional difference in the functional independence rate.

Although access to EVT treatment is available round the clock in many developed countries, many institutions in the United Kingdom and various parts of the world have limited EVT capacity. Furthermore, long distance transfers and travel times, for example, in Australia, have meant some patients may arrive at an EVT‐capable center beyond 24 hours from LKW, but remain eligible for EVT treatment.^18^, ^19^ A large proportion of patients deemed eligible for EVT in our study had a moderate to good collateral supply, which may suggest the presence of a suitable penumbral profile and a limited infarct core. A high rate of successful reperfusion (95%) was also demonstrated in our study among patients treated with EVT, contrary to previous reports which suggested that the probability of successful reperfusion decreases to as low as 42% at 24 hours, possibly due to evolving clot composition over time.^20^, ^21^ Hence, EVT treatment should be considered in carefully selected patients, while a simplified imaging based patient selection process for EVT using NCCT/CTA alone to estimate the infarct size (ASPECTS) and collateral supply irrespective of the time window may be utilized.

The main strength of this study is that EVT service constraints in UK practice created 2 cohorts of patients by default rather than selection, which allowed a comparison of 2 treatment approaches between equivalent study populations without physician influence with regards to treatment allocation. This contention is supported by the fact that the policy of no treatment outside the established hours was pursued without exception and minimizes selection bias, a major confounder in real‐world retrospective analyses comparing EVT with BMM for large vessel occlusion stroke. There are several limitations of this study. First, due to the scarcity of patients presenting in this time period and hence the small sample size in each group, our analyses are likely to have been underpowered and should be interpreted with caution. Second, due to its observational design, confounding by indication and selection bias of a few patients (n=4) in the EVT group who presented outside working hours but remained eligible the next day following repeat neuroimaging and were treated with EVT may have influenced the results. It is also possible that differences in the outcomes may be due to the inherent variances in the patient populations with large vessel occlusion stroke presenting during regular hours versus outside of regular hours, with the potential for inferiority of care for patients admitted outside regular hours due to human diurnal rhythms. This possibility should also be considered in the sample size power analysis for a potential future trial which would randomize patients irrespective of the time of presentation. However, to the best of our knowledge, we are not aware of studies demonstrating such differences. Third, there were some differences in between‐group baseline characteristics likely attributable to the small number of patients in both groups and the eligible patients treated with EVT the next day following repeat neuroimaging. To mitigate confounding, baseline variables of clinical relevance were adjusted for in multivariable analyses. Fourth, a large proportion of patients had an unwitnessed stroke onset, which may have overestimated the duration of stroke symptoms in our study population. Fifth, a small group (n=6) of patients did not undergo CTA imaging on arrival precluding accurate assessment of the clot location and collateral circulation in the BMM cohort, although baseline NCCT imaging with an evident hyperdense vessel sign were retrospectively assessed by trained neuroradiologists. Lastly, the outcome measures, although collated prospectively, were not independently evaluated by a core laboratory.

## Conclusion

In this study, EVT beyond 24 hours from LKW appears safe and feasible, without any significant increase in safety outcomes of symptomatic intracranial hemorrhage or mortality, when performed in patients with acute ischemic stroke who were deemed eligible for EVT by NCCT/CTA alone. A numerically higher number of patients treated with EVT achieved functional independence, compared with treatment with BMM only. However, due to the scarcity of patients presenting in this time period, the overall numbers were too small to reliably comment on the efficacy of EVT. A large collaborative randomized trial assessing the efficacy of EVT beyond 24 hours is warranted. Our findings provide a basis for the sample size estimate for an adequately powered trial.

## Author Contributions

Conception and design, acquisition of the data: Permesh Singh Dhillon. Analysis and interpretation of the data: Permesh Singh Dhillon, Waleed Butt. Critical revision of the manuscript: Permesh Singh Dhillon, Waleed Butt, Tudor G. Jovin, Anna Podlasek, Norman McConachie, Robert Lenthall, Sujit Nair, Luqman Malik, Kailash Krishnan, Robert A. Dineen, and Timothy J. England. Study supervision: Robert A. Dineen, Timothy J. England. All authors approved the final version of the manuscript.

## Sources of Funding

No specific funding was sought for this study.

## Disclosures

Tudor G. Jovin is advisor and investor for Anaconda, Route92, Viz.AI, FreeOx, Blockade Medical, and Methinks. He received personal fees in his role on the Data Safety Monitoring Board and steering committee from Cerenovus and on the screening committee for Contego Medical. He received stock as an advisory board member for Corindus. He received grant support from Medtronic and from Stryker Neurovascular in his capacity as principal investigator for DAWN and AURORA. No other disclosures or competing interests declared by the remaining authors.
